# Real-World Analysis of the Therapeutic Management and Disease Burden in Chronic Myeloid Leukemia Patients with Later Lines in Italy

**DOI:** 10.3390/jcm11133597

**Published:** 2022-06-22

**Authors:** Massimo Breccia, Francesca Chiodi, Aurelio Pio Nardozza, Diletta Valsecchi, Valentina Perrone, Diego Sangiorgi, Elisa Giacomini, Maria Chiara Rendace, Paola Coco, Eleonora Premoli, Luca Degli Esposti

**Affiliations:** 1 Hematology, Department of Translational and Precision Medicine, Sapienza University Policlinico Umberto I, 00161 Rome, Italy; breccia@bce.uniroma1.it; 2 Novartis Farma S.p.A., 21042 Origgio, Italy; francesca.chiodi@novartis.com (F.C.); aurelio_pio.nardozza@novartis.com (A.P.N.); diletta.valsecchi@novartis.com (D.V.); maria_chiara.rendace@novartis.com (M.C.R.); paola.coco@novartis.com (P.C.); eleonora.premoli@novartis.com (E.P.); 3 CliCon S.R.L. Società Benefit, 40137 Bologna, Italy; valentina.perrone@clicon.it (V.P.); diego.sangiorgi@clicon.it (D.S.); elisa.giacomini@clicon.it (E.G.)

**Keywords:** CML, real-life, tyrosine kinase inhibitors, second line TKI, oncology clinical practice

## Abstract

**Simple Summary:**

Real world data represent a useful tool to obtain understanding into the management of cancer disease in routine daily practice. To date, little is known on management and burden of later lines for chronic myeloid leukemia (CML) treatment in Italy. Therefore, we conducted a real-world study to evaluate the characteristics, treatment pattern and drug utilization of patients with CML in 2 or ≥3 tyrosine kinase inhibitor (TKI) lines of therapy to estimate the impact of disease burden. Findings from our study underline an increasing complex management of CML patients while moving on later lines and suggest that the availability of more therapeutic options for CML patients might be an existing need.

**Abstract:**

Real world data are becoming a crucial tool to understand how cancer is treated in routine daily practice. This real-world analysis aims to describe the characteristics of patients with CML in 2nd or ≥3rd tyrosine kinase inhibitors (TKI) lines of therapy, to evaluate their treatment sequence and utilization in settings of Italian clinical practice in Italy. A retrospective analysis was performed using an administrative databases covering around 15.3 million cases. All adult patients prescribed with TKI as 2nd or ≥3rd lines (L) of therapy for CML during January 2015–December 2018 were included. A total of 491 patients in 2nd and 144 in ≥3rd L was included. In both cohorts, hypertension was the most reported comorbidity, followed by metabolic and blood count alterations. In each calendar inclusion year, an increment of 97.6% was observed in the number of patients treated in ≥3rd L. In the 2nd L cohort, 18.7% had a switch to 3rd L, while 26.4% of ≥3rd L patients switched to a subsequent line. Around 40% in both lines discontinued their treatment after a median time of 5.5 (2nd L) and 4.3 (≥3rd L) years. The results provided insights into CML management clinical practice, indicating a heavy disease burden for patients in later lines that showed an increasing complex management, and suggest that a need for novel treatment strategies might exists.

## 1. Introduction

Chronic myeloid leukemia (CML) is a myeloproliferative neoplasm characterized by uncontrolled proliferation of mature granulocytes and by a reciprocal balanced chromosomal translocation between the long arms of chromosome 9 and 22. The resulting chromosome 22, known as Philadelphia chromosome, originates the BCR-ABL gene 1 which encodes a fusion protein with constitutive kinase activity [1,2].

CML treatment usually starts soon after the confirmation of cytogenetic and molecular features. The cornerstone of CML treatment is represented by drugs that specifically target the BCR-ABL ATP-binding site, named tyrosine kinase inhibitors (TKIs), which have transformed CML from a deadly to a chronic and manageable disease [3].

To date, several TKIs have been approved for the treatment of CML. The choice is based on a patient-centered approach, determined by considering the treatment goals, age, comorbidities and the drug-related early and late toxicity [3,4]. According to the 2020 European Leukemia Net recommendations [3], the TKIs that can be used as first-line or second-line of therapy are represented by imatinib (first-generation TKI), bosutinib, dasatinib and nilotinib (second generation TKIs) [3,5,6,7]. The third-generation TKI ponatinib is approved for second and later lines and for patients with T315I mutation [8].

Nonetheless there are guidelines and recommendations that help physicians in the choice of the first line and subsequent lines of therapies; clinical judgment remains the cornerstone of therapy’s choice [1,3,9].

In oncology, the generation of real-world evidence to answer clinical and policy-relevant questions that cannot be directly or completely answered using data from randomized clinical trials (RCTs) has rapidly gained increased interest in recent years [4,10]. Indeed, real-world data represent an important opportunity for the research community to gain valuable insights into the management of the disease and patient outcomes in routine daily oncology practice, without the possible selection bias included in RCTs.

Little evidence is available so far regarding the CML scenario in Italian clinical practice in terms of therapeutic management and burden on the disease. By querying administrative databases, we profiled and evaluated the characteristic and co-morbidity features of a CML population treated in 2nd or later lines in Italy, their drug utilization and therapeutic sequence, providing for the first time a comprehensive, real-word, country-specific snapshot of CML management in later lines in settings of Italian clinical practice.

## 2. Materials and Methods

### 2.1. Study Design and Data Source

An observational retrospective analysis was carried out by integrating administrative databases from a sample of Italian Local Health Units (LHUs), based on a sample of 15 million inhabitants (around 25% of the Italian population) across Italy. These databases hold information on healthcare resources consumption reimbursed by the Italian National Health Service (INHS).

The following databases have been queried: beneficiaries’ database, which contains all demographic data for patients in analysis; pharmaceuticals database, which contains the data on the drugs supplies for patients in analysis as anatomical therapeutic code (ATC), prescription date, number of packages; hospitalization database, which contains all hospitalization data for patients in analysis as date of hospitalization, main and secondary diagnosis identified by International Classification of Diseases, Ninth Revision, Clinical Modification (ICD-9-CM), Diagnosis Related Group (DRG); laboratory tests and specialist visits database, which contains data on the type of laboratory test or specialist visit and prescription date.

To guarantee patient privacy, an anonymous univocal numeric code (Patient ID) was assigned to each health-assisted subject by the LHUs. The Patient ID code allowed electronic linkage between databases. The anonymous code of the patient ensures the anonymity of the extracted data in full compliance with UE Data Privacy Regulation 2016/679 (“GDPR”) and Italian D.lgs. n. 196/2003, as amended by D.lgs. n. 101/2018. All the results of the analyses were produced as aggregated summaries, which could not be connected, either directly or indirectly, to individual patients.

### 2.2. Study Periods

The study periods for each patient were defined as described below. The data availability period went from 1 January 2010 to 31 December 2018. During this time, the inclusion period was selected based on the availability in Italy of all the TKI in analysis and was from 1 January 2015 to 31 December 2018. The index date corresponded to the date of the first TKI prescription in 2nd or ≥3rd lines of therapy. The pre-index period regarded all periods before the index date and was used to characterize patients. The post-index period was from index date until the end of study period, date of death or exiting the database (whatever occurred first).

### 2.3. Study Population

Considering that CML patients are normally followed during outpatient visits, the identification criteria considered were mainly based on the prescription of specific therapies for CML. The therapies present in the therapeutic panorama are either prescribed only for CML—and therefore directly attributable—or non-specific. In the latter case, to identify patient with CML, additional criteria were used. Specifically, all patients ≥ 18 years of age were screened for inclusion if they presented at least one prescription for TKIs A bosutinib, dasatinib, imatinib, nilotinib, ponatinib. In the absence of a prescription of nilotinib or bosutinib, specific for CML only, to identify those patients having CML and no other TKI-treated medical conditions, the following additional inclusion criteria were applied: at least one hospitalization (day hospital or regular admission) with main or secondary discharge diagnosis of CML or at least one prescription for BCR-ABL exam without hospitalization (day hospital or regular admission) with main or secondary discharge diagnosis of lymphoid acute leukemia during the entire study period. A list of specific codes used were reported in Table S1.

Among all patients identified by the abovementioned criteria, only those in 2nd L and ≥3rd lines of TKI treatment during the identification period were included in the study. All patients included in the study were stratified in two not mutually exclusive cohorts according to the number of lines of therapy, namely 2nd L (patients in 2nd line) and ≥3rd L (patients in ≥3rd line) cohorts.

### 2.4. Study Variables

Presence of comorbidities was considered in all available pre-index periods (i.e., the first TKI prescription in 2nd or ≥3rd lines of therapy) and post-index during the treatment. To identify the comorbidities listed in Table S2, both primary and secondary diagnoses collected in the hospitalization database were considered. In order to minimize potential underestimation, especially for diagnosis not frequently reported in hospital settings, the comorbidities were also identified using as proxy the therapies prescribed to the patient. Comorbidities were reported as number and % of patients with a certain comorbidity among all patients and within each TKI group.

The number of lines of the TKI in analysis was determined by looking at prescription for TKIs during all data periods available for the study (2010–2018). All analyses were performed considering all patients in 2nd L (i.e., with a previous TKI) and ≥3rd L (with at least two previous TKIs) overall and stratified by type of TKI in analysis. The number of lines were also reported for each year of the identification period (2015–2016–2017–2018). The number of patients incident to line in each year of inclusion not necessarily correspond to the patients incident to specific TKI during all inclusion periods.

The following variables were investigated and reported as number and percentages of patients within each year: incident to line (as in patients that started the 2nd or 3rd lines within the calendar year); switch to subsequent line (as in patients changing TKI thus moving towards lines of therapy within each calendar year); interruptions (as in patients without TKI prescription in the following year); deaths within each calendar year. Because of data privacy, only treatment sequences involving ≥4 patients were reported. Moreover, the mean daily dosage was evaluated in both cohorts of patients presenting at least two prescriptions and calculated as the total dosage between first and last prescription divided per the total number of days between first and coverage of last prescriptions.

Kaplan Meier method was applied to analyze the time to discontinuation in patients with 2nd L or ≥3rd L as time (in months) from therapy start to permanent discontinuation (plus last prescription duration for alive patients, date of death plus one day for dead patients). If a patient was still on treatment at the end of database availability period, he/she was censored at the date of end of database availability.

All the analyses were performed as descriptive and on two different cohorts of patients in analysis. According to “Opinion 05/2014 on Anonymisation Techniques” drafted by the “European Commission Article 29 Working Party”, the analyses involving less than three patients were not reported, as potentially reconductable to single individuals. Therefore, results that referred to ≤3 patients were not reported (NR, not reported). All analyses were performed using STATA SE, version 12.0.

## 3. Results

### 3.1. Baseline Characteristics

Overall, 491 patients were identified and included in the 2nd L cohort and 144 patients in ≥3rd L cohort (Table 1). In the 2nd L cohort, 201 patients (mean age 60.3 years, 59.2% male) received dasatinib, 142 (mean age 60.4 years, 52.1% male) nilotinib, 60 (mean age 68.2 years, 53.3% male) bosutinib, 50 (mean age 56.1 years, 64% male) ponatinib and 38 (mean age 63.0 years, 65.8% male) imatinib (Table 1a). At baseline, hypertension was the most reported comorbidity (91.7% bosutinib, 86.8% imatinib, 68.0% ponatinib, 65.2% dasatinib, 64.1% nilotinib), followed by metabolic (63.2% imatinib, 55.0% bosutinib, 33.8% nilotinib, 32.0% ponatinib, 27.9% dasatinib) and blood count (58.0% ponatinib, 48.3% bosutinib, 34.2% imatinib, 33.1% nilotinib, 28.4% dasatinib) alterations. Cardiovascular conditions affected around 23% of patients in 2nd L (46.7% bosutinib, 36.8% imatinib, 19.7% nilotinib, 18.0% ponatinib, 16.4% dasatinib). In the ≥3rd L, 38 patients (mean age 64.6 years, 42.1% male) were treated with imatinib, 32 (mean age 64.8 years, 40.6% male) with ponatinib, 27 (mean age 57.0 years, 44.4% male) with nilotinib, 24 (mean age 69.5 years, 62.5% male) with bosutinib and 23 (mean age 63.4 years, 60.9% male) with dasatinib (Table 1b). The trend of comorbidities was similar to that reported for second line treatment, with hypertension detected in all patients starting bosutinib, 81.3% in those prescribed ponatinib, 78.9% imatinib, 70.4% nilotinib and 65.2% dasatinib, followed by blood count alteration (59.4% ponatinib, 58.3% bosutinib, 48.1% nilotinib, 39.5% imatinib, 34.8% dasatinib) and metabolic alterations (50.0% bosutinib, 47.8% dasatinib, 47.4% imatinib, 37.5% ponatinib, 37.0% nilotinib). Moreover, the occurrence of comorbidities in 2nd L and ≥3rd L cohorts assessed during the treatment is reported in Tables S3 and S4. Specifically, hypertension occurred in 5.5% and 6.3% in 2nd L and ≥3rd L cohorts, respectively, and cardiovascular comorbidities in 5.7% and 3.5% of patients in 2nd L and ≥3rd L, respectively.

### 3.2. Treatment Patterns and Drug Utilization

In each calendar year from 2015 to 2018, the incidence of patients who entered a 2nd L in each year was 22.6% for year 2015, 28.7% for year 2016, 24.9% for year 2017 and 23.5% in 2018, whereas higher values were reported for incidence of patients starting a ≥3rd L of treatment (43.9% in 2015, 46.7% in 2016 with a decrement in the following years 2017 and 2018, in which incident patients were 38.7% and 37.0%, respectively) (Table 2). For patients in 2nd L, switch to subsequent line was constant during each calendar year and was reported within the range 6.2% (2017)–7.0% (2018). An increasing trend in the number of patients treated in ≥3rd L cohort over the year was observed, with a 97.6% increment over the index date (2015–2018) (from 41 patients in 2015 to 81 in 2018, Table 2).

**Figure 1:**
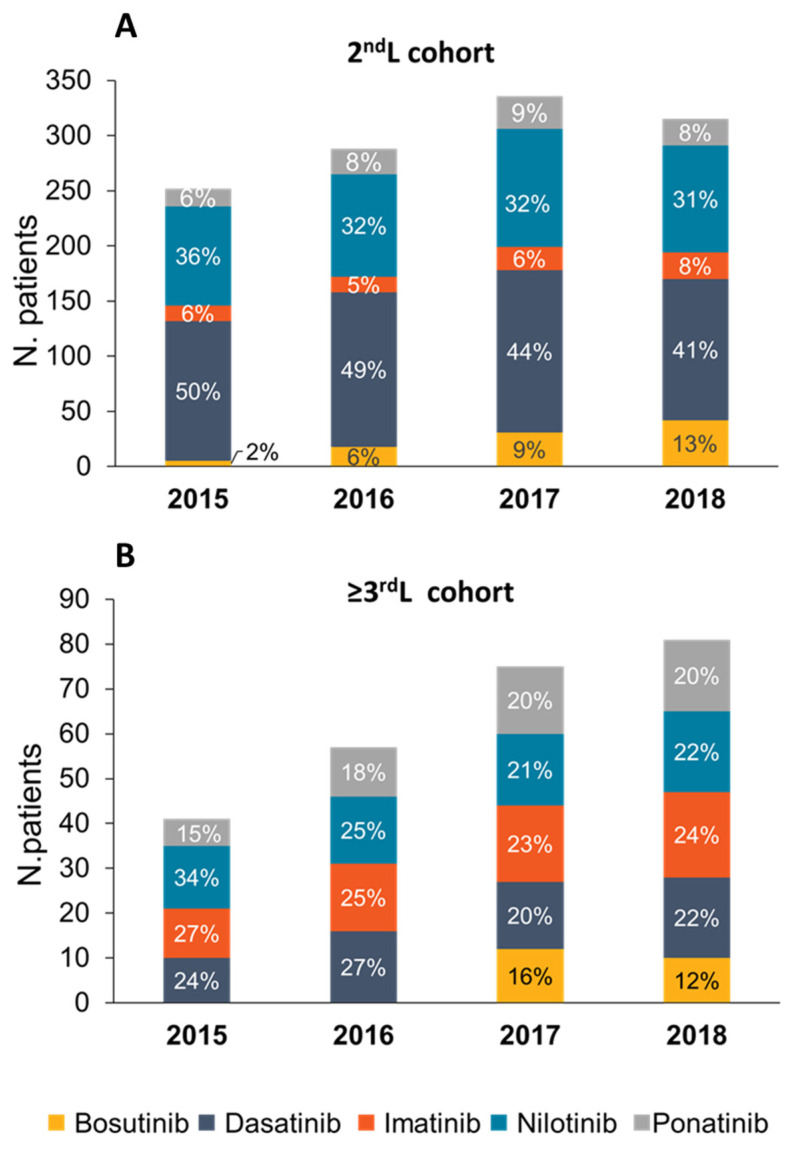
Distribution of patients by TKI in each calendar year in the 2nd L (**A**) and ≥3rd L (**B**).

In terms of TKI chosen, bosutinib showed a linear increase (from 2% to 13%) over the years, while dasatinib and nilotinib showed an opposite trend (dasatinib decreased from 50% in 2015 to 41% in 2018 and nilotinib decreased from 36% in 2015 to 31% in 2018). Constant utilization of imatinib and ponatinib was observed over the years (Figure 1A). Among patients with ≥3rd line (Figure 1B), aside from ponatinib prescription (increased from 15% in 2015 to 20% in 2018) and imatinib (no substantial changes over the years), nilotinib, dasatinib and bosutinib did not show a clear trend.

In patients in 2nd or to ≥3rd L of treatment, respectively, the daily dosage of the index molecule averaged 340.7 and 382.2 mg/die for bosutinib, 92.7 and 81.4 mg/die for dasatinib, 395.5 and 481.5 mg/die for imatinib, 609.2 and 656.4 mg/die for nilotinib, and 31.8 and 37.9 mg/die for ponatinib (Table S5).

Among patients in analysis, incidence to 2nd or to ≥3rd L of treatment was reported to be 57.8% and 56.9%, respectively (Table 3) (non-incident patients were already in that specific line of treatment). In the 2nd L cohort, 18.7% of patients switched to 3rd L (ranging from 14.1% in nilotinib group to 25% in bosutinib group), 3.5% had more than one switch and moved to fourth or more lines. Patients still in treatment at the end of follow-up were distributed as follows: 62.7% nilotinib (mean follow-up 3.3 years), 55.7% dasatinib (mean follow-up 3.2 years), 53.3% bosutinib (mean follow-up 2.1 years), 50.0% ponatinib (mean follow-up 2.1 years), 47.4% imatinib (mean follow-up 2.7 years) (Table 3a). In the ≥3rd L cohort, 26.4% had a switch to a subsequent line, while patients still in treatment at the end of follow-up were 55.3% in the imatinib group (mean follow-up 2.8 years), 51.9% nilotinib (mean follow-up 3.3 years), 50.0% bosutinib (mean follow-up 1.6 years), 40.6% ponatinib (mean follow-up 2.2 years), 34.8% dasatinib (mean follow-up 3.7 years).

**Figure 2:**
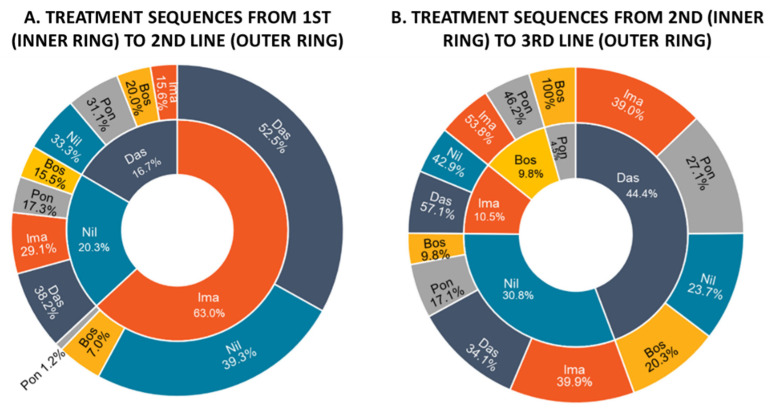
Treatment sequences from 1st (inner ring) to 2nd line (outer ring) (**A**) and from 2nd (inner ring) to 3rd line (outer ring) (**B**) considering all patients included in the analysis. Abbreviations: Bos, bosutinib; Das, dasatinib; Ima, imatinib; Nil, nilotinib; Pon, ponatinib.

The analysis of the treatment sequences showed that patients starting with dasatinib as 1st L switched more frequently to nilotinib (33.3%) and ponatinib (31.1%) as 2nd L. The remaining third, was almost equally shared between bosutinib and imatinib (20% and 15.6%, respectively). More than half of patients (52.5%) who started with imatinib as 1st line had dasatinib as 2nd L, followed by nilotinib (39.3%) and bosutinib (7%) and ponatinib (1.2%). Patients with nilotinib as 1st switched to dasatinib (38%), imatinib (29%), ponatinib (17.3%) and bosutinib (15.5%) as 2nd L (Figure 2A). Patients with bosutinib as 2nd L switched either to imatinib (54%) or ponatinib (46%) as 3rd L. Patients with imatinib as 2nd L had instead either dasatinib (57,1%) or nilotinib (42,9%) as 3rd L. The switch pattern of patients starting either with dasatinib or nilotinib as 2nd L is more heterogeneous. Specifically, the 39% of patients who had dasatinib in second L switched to imatinib, 27.1% to ponatinib, 23,7% to nilotinib and 20,3% to bosutinib. Patients who had nilotinib as 2nd L switched to imatinib (39.9%) followed by dasatinib (34.1%), ponatinib (17.1%) and bosutinib (9.8%) as 3rd L. Notably, all patients who were treated with ponatinib in 2nd L switched to bosutinib as 3rd L (Figure 2B).

**Figure 3:**
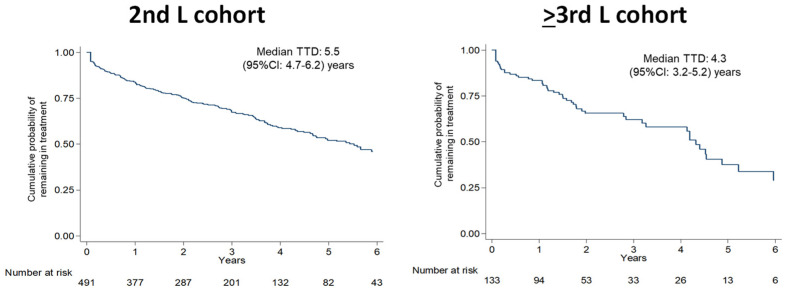
Time to discontinuation.

After a median follow-up of 3.0 and 2.6 years, the proportion of deceased patients was 13.2% and 19.4% in 2nd L and ≥3rd L cohorts, respectively (Table 3). Around 40% in both the cohorts, discontinued their treatment, with a median time to discontinuation of 5.5 (95%CI: 4.7–6.2) years in the 2nd L cohort and 4.3 (95%CI: 3.2–5.2) years in the ≥3rd L cohort (Figure 3).

The management and health care impact information of later lines treatment can be deduced from Table 4 using, as readout, the number of all-cause visits and all-cause hospitalizations. Regardless the TKI used, overall, the 2nd L and ≥3rd L cohorts show a similar impact, however, some differences both over TKIs and line of therapy emerge. In terms of visits, in the 2nd L cohort, imatinib shows the lowest mean value (5.4) followed by nilotinib and dasatinib (6.6), ponatinib (8.3) and bosutinib (8.6) while, in ≥3rd L cohort nilotinib shows the lowest mean values (4.5) followed by imatinib (5.1) and bosutinib (7.4), dasatinib (8.0) and ponatinib (8.4). The mean annual number of hospitalizations in 2nd L cohort shows nilotinib has the lowest (0.4) and ponatinib as highest (1.1). In ≥3rd L cohort imatinib and nilotinib show equal mean annual number of hospitalization (0.4), followed by dasatinib and bosutinib (0.5) and ponatinib (0.7).

## 4. Discussion

To date, treatment strategy of later lines is poorly established and limited options are currently available for CML patients that fail 2nd L of therapy [3,11]. Real-world studies in the oncology field could produce useful data in terms of treatment sequences and therapeutic pathways on patients evaluated out of the strict criteria required from RCTs. Furthermore, real-world evidence may give insights into the quality of care and provide perspective of the present and future scenarios for this disease in clinical practice. The study presented herein fits in this context since it provided a demographic and clinical profile of CML patients in 2nd and ≥3rd TKI lines and described drug utilization in real-world setting of the Italian clinical practice.

The long-term treatment strategy required to manage the chronic course of the disease should depend on demographic characteristics as patient’s age, especially for older patients that could be more likely to present with comorbidities and subsequently concomitant medications [12]. The analysis of the patients’ characteristics showed that the comorbidity profile was in line with the age trend and that bosutinib tends to be prescribed to older patients in both 2nd L and ≥3rd L cohorts. In this direction, RCTs are currently ongoing [13] on the use of bosutinib in older population, and real-life studies evaluated the efficacy of this drug on elderly patients with intolerance/resistance to other TKIs [14], suggesting an increased use of bosutinib in this sub-population. This tendency could also explain the switch to bosutinib as 3rd L observed in the analysis of line sequence.

Interestingly, while the distribution of patients by type of 2nd TKI prescribed was in line with other studies reporting nilotinib and dasatinib as the most frequently prescribed for patients in 2nd L [15,16], a variability in terms of TKI prescribed in patients in their ≥3rd L was observed. Dahlen and colleagues [17] reported a different pattern for patients with ≥3rd L in three European registries, with nilotinib being most commonly prescribed followed by dasatinib and bosutinib, although high variability was detected between the analyzed countries. The abovementioned differences could be explained by: (i) the different time periods observed (from 2008 vs. 2015 in our study), (ii) the lack of specific guidelines/indication in later lines setting as well as (iii) by the country-specific clinical practice. Patients with imatinib as 3rd L could be due to imatinib rechallenge for relapsing patients [18]. To the best of our knowledge, there are no similar studies evaluating how in Italy TKIs are distributed in later lines of treatment, providing a real word overview of the therapeutic strategy Italian physicians adopt in their clinical practice.

An upward trend of patients treated with TKIs in ≥3rd L over the years was observed, suggesting the complexity of CML management. The distribution of prescriptions within each inclusion year had few fluctuations, which should, however, be interpreted considering the low number of patients in some treatment groups. Around 6–7% of 2nd L patients were reported to switch to a 3rd L each year, yielding a switch rate of around 19% in the overall period. A sustained trend of switch of therapy was also reported in almost one-fourth of patients in ≥3rd L cohort. An elevated switch rate was reported in an American retrospective study as well, with nearly 1 in every 3 patients moving from 2nd to 3rd L and around 27% of patients moving from 3rd to 4th L [15].

Consistently, we detected a high rate of treatment discontinuation (around 40%) in both cohorts. As for the 3rd L cohort, our results were comparable with another retrospective study among the German population in which 21 out of 53 (~40%) CML patients in 3rd L discontinued treatment [19]. Moreover, data from the phase 4 BYOND study on bosutinib-treated patients showed a discontinuation rate averaging almost 44%, specifically 33%, 46% and 51% in patients in 2L, 3L and 4L, respectively [20]. Data from randomized clinical trials showed that the discontinuation rate for TKI is around 40–50% [21] (the months were slightly higher compared to TKI discontinuation rate we found in our analysis), with a median duration on TKI ranging 43.5–90 months [21]. Despite being of great interest, the reason why we observed a sustained trend of switch cannot be either extracted or inferred for this analysis. However, our data highlight that the availability of more therapeutic options for CML patients might be an existing need.

The management of CML patients who have failed one or more lines of therapy remains a critical issues handle. Life expectancy for CML patients in the chronic phase, if treated, is now similar to that of the general population [22]. However, this is no one size fits all statement. Indeed, we know from the literature that long life TKI therapy and later lines suggest a significant increase in the complexity in the management of CML patients [23]. Despite 1 L annual mean numbers of visits and hospitalizations are missing in our analysis, it seems clear that later lines, in absolute value, have a significant burden for both patients, physicians and health care systems, suggesting that later line CML management, with the current available treatments, is still an important need to address.

The study has limitations: our cohort of patients reflected real clinical practice, and the results must be interpreted considering the limitations related to the observational nature of the study, based on data collected through administrative databases. The first limitation is represented by the potential underestimation of the sample population since it was not possible to collect data for patients in clinical trials. Secondly, we acknowledge the lack of clinical information, such as data related to the severity or to the CML phase, the reasons and causes to move therapies forward, to discontinue therapy or to switch therapy, which are not reported within the databases. In this regard, administrative databases do not store information on blastic crisis or on disease acceleration, therefore we could have overestimated the patients in chronic phase. Response to treatment or results concerning mutational analyses were not provided as well. Although we do recognize the importance of such crucial clinical information, the focus of the analyses was to provide a snapshot on how CML patients with later lines are managed in clinical practice and was not intended to assess patients’ outcomes. Since the comorbidities herein analyzed were addressed based on available data before or after inclusion (using proxy of diagnosis, such hospitalization discharge diagnosis and/or specific treatments), there might be an incomplete capture of these comorbidities among patients. Moreover, the results must be interpreted considering the low sample size in some treatment groups, especially in the 3rd L cohort. Ultimately, the results of this study are limited to the population analyzed and may not be generalizable to the entire national population.

## 5. Conclusions

In conclusion, this real-world study underlined a heavy clinical burden for patients in later lines, especially in terms of comorbidities, treatment discontinuation and rising proportion of patients with multiple lines that may indicate an increasingly complex CML management. In line with characteristics observed, in both cohorts a similar use of healthcare resource consumptions was reported. Overall, our findings depict a real-word snapshot of Italian CML patients and CML clinical practice in later lines and could represent a useful tool to inform health-policy makers on how these patients are currently managed. Furthermore, our results could suggest the need of novel therapeutic options and/or treatment strategies for the management of later lines of CML.

## Figures and Tables

**Table 1:** Baseline characteristics of patients in 2nd L (**a**) and ≥3rd L (**b**) cohorts stratified by TKI agents.

**(a) 2nd L Cohort**	**Bosutinib**	**Dasatinib**	**Imatinib**	**Nilotinib**	**Ponatinib**	**Overall**
N. of patients	60	201	38	142	50	491
Age (mean, SD)	68.2 (10.4)	60.3 (14.3)	63.0 (15.6)	60.4 (15.4)	56.1 (16.3)	61.1 (14.8)
Male (n, %)	32 (53.3)	119 (59.2)	25 (65.8)	74 (52.1)	32 (64.0)	282 (57.4)
Comorbidities ^1^						
Hypertension (n, %)	55 (91.7)	131 (65.2)	33 (86.8)	91 (64.1)	34 (68.0)	344 (70.1)
Cardiovascular (n, %)	28 (46.7)	33 (16.4)	14 (36.8)	28 (19.7)	9 (18.0)	112 (22.8)
Ischemic heart disease	10 (16.7)	20 (10.0)	8 (21.1)	11 (7.7)	5 (10.0)	54 (11.0)
Diseases of pulmonary circulation	0 (0.0)	0 (0.0)	NR	NR	0 (0.0)	NR
Other forms of heart disease	17 (28.3)	14 (7.0)	8 (21.1)	18 (12.7)	5 (10.0)	62 (12.6)
Cerebrovascular disease	13 (21.7)	11 (5.5)	7 (18.4)	8 (5.6)	NR	42 (8.6)
Diseases of veins and lymphatics	NR	NR	0 (0.0)	NR	NR	7 (1.4)
Pneumonia and pleurisy (n, %)	13 (21.7)	12 (6.0)	NR	23 (16.2)	15 (30.0)	66 (13.4)
Pneumonia and influenza	NR	NR	NR	7 (4.9)	6 (12.0)	18 (3.7)
COPD and allied condition	NR	5 (2.5)	0 (0.0)	7 (4.9)	5 (10.0)	20 (4.1)
Other diseases respiratory system	10 (16.7)	8 (4.0)	NR	17 (12.0)	7 (14.0)	45 (9.2)
Gastrointestinal (n, %)	5 (8.3)	15 (7.5)	NR	11 (7.7)	6 (12.0)	40 (8.1)
Liver (n, %)	NR	12 (6.0)	0 (0.0)	5 (3.5)	4 (8.0)	24 (4.9)
Renal (n, %)	4 (6.7)	5 (2.5)	4 (10.5)	4 (2.8)	6 (12.0)	23 (4.7)
Edema (n, %)	0 (0.0)	0 (0.0)	0 (0.0)	0 (0.0)	0 (0.0)	0 (0.0)
Blood count alterations (n, %)	29 (48.3)	57 (28.4)	13 (34.2)	47 (33.1)	29 (58.0)	175 (35.6)
Metabolic alterations (n, %)	33 (55.0)	56 (27.9)	24 (63.2)	48 (33.8)	16 (32.0)	177 (36.0)
Pre-index period, years (mean, SD)	6.3 (1.9)	5.6 (1.6)	5.6 (2.0)	5.5 (1.8)	5.5 (1.9)	5.7 (1.8)
**(b) ≥3rd L Cohort**	**Bosutinib**	**Dasatinib**	**Imatinib**	**Nilotinib**	**Ponatinib**	**Overall**
N. of patients	24	23	38	27	32	144
Age (mean, SD)	69.5 (9.8)	63.4 (16.6)	64.6 (12.5)	57.0 (14.7)	64.8 (12.9)	63.8 (13.7)
Male (n, %)	15 (62.5)	14 (60.9)	16 (42.1)	12 (44.4)	13 (40.6)	70 (48.6)
Comorbidities ^1^						
Hypertension (n, %)	24 (100.0)	15 (65.2)	30 (78.9)	19 (70.4)	26 (81.3)	114 (79.2)
Cardiovascular (n, %)	13 (54.2)	6 (26.1)	14 (36.8)	6 (22.2)	12 (37.5)	51 (35.4)
Ischemic heart disease	7 (29.2)	NR	6 (15.8)	NR	4 (12.5)	22 (15.3)
Diseases of pulmonary circulation	0 (0.0)	NR	0 (0.0)	NR	NR	NR
Other forms of heart disease	8 (33.3)	NR	10 (26.3)	NR	9 (28.1)	31 (21.5)
Cerebrovascular disease	5 (20.8)	NR	5 (13.2)	NR	4 (12.5)	17 (11.8)
Diseases of veins and lymphatics	NR	0 (0.0)	0 (0.0)	NR	NR	NR
Pneumonia and pleurisy (n, %)	11 (45.8)	4 (17.4)	10 (26.3)	6 (22.2)	5 (15.6)	36 (25.0)
Pneumonia and influenza	NR	NR	0 (0.0)	NR	NR	10 (6.9)
COPD and allied conditions	NR	0 (0.0)	4 (10.5)	NR	NR	10 (6.9)
Other diseases respiratory system	8 (33.3)	NR	9 (23.7)	4 (14.8)	NR	26 (18.1)
Gastrointestinal (n, %)	NR	NR	4 (10.5)	NR	NR	15 (10.4)
Liver (n, %)	4 (16.7)	0 (0.0)	4 (10.5)	NR	NR	11 (7.6)
Renal (n, %)	4 (16.7)	0 (0.0)	NR	NR	NR	11 (7.6)
Edema (n, %)	0 (0.0)	0 (0.0)	0 (0.0)	0 (0.0)	NR	NR
Blood count alterations (n, %)	14 (58.3)	8 (34.8)	15 (39.5)	13 (48.1)	19 (59.4)	69 (47.9)
Metabolic alterations (n, %)	12 (50.0)	11 (47.8)	18 (47.4)	10 (37.0)	12 (37.5)	63 (43.8)
Pre-index period, years (mean, SD)	6.6 (2.3)	5.0 (1.9)	5.8 (1.5)	5.0 (2.0)	5.8 (1.7)	5.7 (1.9)

^1^ All available backward period. Note. According to “Opinion 05/2014 on Anonymisation Techniques” drafted by the “European Commission Article 29 Working Party”, the analyses involving less than three patients were not reported, as potentially reconductable to single individuals. Therefore, results referred to ≤3 patients were not reported (NR, not reported).

**Table 2:** Treatment patterns per calendar year of identification period (2015–2018).

**2nd L Cohort**	**2015**	**2016**	**2017**	**2018**
N. of patients	252	288	336	315
Incident to line (n, %)	57 (22.6)	83 (28.7)	84 (24.9)	74 (23.5)
Switch to subsequent line, same year (n, %)	17 (6.7)	19 (6.6)	21 (6.2)	22 (7.0)
Death, same year (n, %)	6 (2.4)	9 (3.1)	10 (3.0)	4 (1.3)
Interruptions (n, %)	24 (9.1)	8 (2.8)	64 (19.0)	-
Follow-up, years (mean, SD)	3.9 (1.2)	3.1 (1.0)	2.2 (0.9)	1.6 (0.5)
**≥3rd L Cohort**	**2015**	**2016**	**2017**	**2018**
N. of patients	41	60	75	81
Incident to line (n, %)	18 (43.9)	28 (46.7)	29 (38.7)	30 (37.0)
Switch to subsequent line, same year (n, %)	NR	4 (6.7)	NR	NR
Death, same year (n, %)	NR	NR	4 (5.3)	6 (7.4)
Interruptions (n, %)	7 (17.1)	8 (13.3)	13 (17.3)	-
Follow-up, years (mean, SD)	3.9 (1.4)	3.4 (1.0)	2.3 (0.9)	1.7 (0.6)

According to “Opinion 05/2014 on Anonymisation Techniques” drafted by the “European Commission Article 29 Working Party”, the analyses involving less than three patients were not reported, as potentially reconductable to single individuals. Therefore, results referred to ≤3 patients were not reported (NR, not reported).

**Table 3:** Treatment patterns of patients in 2nd L (**a**) and ≥3rd L (**b**) cohorts, overall and stratified by TKI agent.

**(a) 2nd L cohort**	**Bosutinib**	**Dasatinib**	**Imatinib**	**Nilotinib**	**Ponatinib**	**Overall**
N. of patients	60	201	38	142	50	491
Incident to 2nd L (n, %)	52 (86.7)	91 (45.3)	23 (60.5)	79 (55.6)	39 (78.0)	284 (57.8)
Follow-up, years (mean, SD)	2.1 (1.1)	3.2 (1.4)	2.7 (1.6)	3.3 (1.5)	2.1 (1.4)	3.0 (1.5)
No switch to 3rd L (n, %)	42 (70.0)	152 (75.6)	30 (78.9)	119 (83.8)	39 (78.0)	382 (77.8)
*Patients still in treatment (n, %)*	32 (53.3%)	112 (55.7%)	18 (47.4%)	89 (62.7%)	25 (50%)	-
Switch to 3rd L (n, %)	15 (25.0)	43 (21.4)	6 (15.8)	20 (14.1)	8 (16.0)	92 (18.7)
Switch to 4th L or more (n, %)	NR	6 (3.0)	NR	NR	NR	17 (3.5)
Death (n, %)	4 (6.7)	27 (13.4)	4 (10.5)	17 (12.0)	13 (26.0)	65 (13.2)
Mean treatment length (years)	1.42	3.21	1.84	3.24	1.50	-
**(b) ≥3rd L cohort**	**Bosutinib**	**Dasatinib**	**Imatinib**	**Nilotinib**	**Ponatinib**	**Overall**
N. of patients	24	23	38	27	32	144
Incident to 3rd L (n, %)	21 (87.5)	10 (43.5)	16 (42.1)	12 (44.4)	23 (71.9)	82 (56.9)
Follow-up, years (mean, SD)	1.6 (1.0)	3.7 (1.5)	2.8 (1.4)	3.3 (1.6)	2.2 (1.4)	2.7 (1.6)
No switch to 4th L (n, %)	19 (79.2)	14 (60.9)	30 (78.9)	17 (63.0)	26 (81.3)	106 (73.6)
*Patients still in treatment (n, %)*	12 (50)	8 (34.8)	21 (55.3)	14 (51.9)	13 (40.6)	-
Switch to 4th L or more (n, %)	5 (20.8)	9 (39.1)	8 (21.1)	10 (37.0)	6 (18.8)	38 (26.4)
Death (n, %)	8 (33.3)	NR	4 (10.5)	4 (14.8)	10 (31.3)	28 (19.4)
Mean treatment length (years)	1.14	2.78	2.42	3.16	1.50	-

Note: According to “Opinion 05/2014 on Anonymisation Techniques” drafted by the “European Commission Article 29 Working Party”, the analyses involving less than three patients were not reported, as potentially reconductable to single individuals. Therefore, results referred to ≤3 patients were not reported (NR, not reported).

**Table 4:** Mean annual number of annual consumptions of healthcare resources of 2nd L (**a**) and ≥3rd L (**b**) cohorts, overall and by type of TKI.

**(a) 2nd L Cohort**	**Bosutinib**	**Dasatinib**	**Imatinib**	**Nilotinib**	**Ponatinib**	**Overall**
N. of patients	60	201	38	142	50	491
All-cause visits (mean, SD)	8.6 (7.9)	6.6 (5.4)	5.4 (5.4)	6.6 (6.6)	8.3 (9.5)	6.9 (6.6)
All-cause hospitalizations (mean, SD)	0.7 (1.3)	0.5 (1.1)	0.5 (1.2)	0.4 (1.0)	1.1 (1.5)	0.6 (1.1)
**(b) ≥3rd L Cohort**	**Bosutinib**	**Dasatinib**	**Imatinib**	**Nilotinib**	**Ponatinib**	**Overall**
N. of patients	24	23	38	27	32	144
All-cause visits (mean, SD)	7.4 (6.7)	8.0 (7.7)	5.1 (4.9)	4.5 (3.5)	8.4 (6.5)	6.6 (6.0)
All-cause hospitalizations (mean, SD)	0.5 (0.9)	0.5 (1.0)	0.4 (1.2)	0.4 (0.7)	0.7 (1.2)	0.5 (1.1)

## Data Availability

All data used for the current study are available upon reasonable request to CliCon s.r.l. which is the body entitled of data treatment and analysis by Local Health Units.

## Supplementary Materials

The following supporting information can be downloaded at: https://www.mdpi.com/article/10.3390/jcm11133597/s1, Table S1: List of codes used for identification of patients included in the analysis. Table S2: List of comorbidities. Table S3: The occurrence of comorbidities during treatment in patients in 2nd L stratified by TKI agents. Table S4: The occurrence of comorbidities during treatment in patients in 3rd L stratified by TKI agents. Table S5: Mean daily dosages.
