# Supplementary material for: Real-World Analysis of the Therapeutic Management and Disease Burden in Chronic Myeloid Leukemia Patients with Later Lines in Italy
Source: J Clin Med. 2022 Jun 22;11(13):3597. doi: 10.3390/jcm11133597 (PMC9267241; doi:10.3390/jcm11133597)
Supplement: Supplementary file 1 [file jcm-11-03597-s001.zip › jcm-1728220-supplementary.pdf]

Supporting Table S1. List of codes used for identification of patients included in the analysis.

**TKI**	**ATC codes in force at the time of study period.**
Bosutinib	L01XE14
Dasatinib	L01XE06
Imatinib	L01XE01
Nilotinib	L01XE08
Ponatinib	L01XE24
**Hospitalization discharge diagnosis/ procedures (*for patients without nilotinib or bosutinib prescriptions*)**	**ICD-9-CM codes**
CML	205.1
BCR-ABL exam (ICD-9-CM code: 91.36.5, 91.29.4, 91.29.3) without hospitalization (day hospital or regular admission) with main or secondary discharge diagnosis of lymphoid acute leukemia	91.36.5, 91.29.4, 91.29.3 without lymphoid acute leukemia (ICD-9-CM code: 204.0)

Supporting Table S2. List of comorbidities.

**Diagnosis**	**ICD-9-CM codes**	**ATC codes**
Hypertension	401-405	C02, C03, C07, C08, C09
Cardiovascular:		
Ischemic heart disease	410-414	
Diseases of pulmonary circulation	415-417	
Other forms of heart disease	420-429	
Cerebrovascular disease, arteries, arterioles, capillaries	430-448	
Diseases of veins and lymphatics	451-455	
Pneumonia and preurisy:		
Pneumonia and influenza	480-487	
COPD and allied conditions	490-496	
Other diseases of respiratory system	510-519	
Gastrointestinal	530-538 /555-558 / 560-569; 787	
Liver	570-579; 790.4	
Renal	580-589; 593.8; 593.9	
Edema	782.3	
Anemia; thromobocytopenia; neutropenia (blood count alterations)	790.0; 280-285; 287.4; 287.5; 288	B03
Metabolic alterations	790.2; 250; 272	A10, C10

Supporting Table S3. The occurrence of comorbidities during treatment in patients in 2nd line stratified by TKI agents

	**Imatinib**	**Dasatinib**	**Nilotinib**	**Bosutinib**	**Ponatinib**	**Overall**
N. of patients	38	201	142	60	50	491
Hypertension (n, %)	0 (0.0)	20 (10.0)	5 (3.5)	0 (0.0)	<4	27 (5.5)
Cardiovascular (n, %)	<4	11 (5.5)	9 (6.3)	<4	<4	28 (5.7)
Ischemic heart disease	<4	4 (2.0)	0 (0.0)	<4	<4	8 (1.6)
Diseases of pulmonary circulation	0 (0.0)	<4	0 (0.0)	<4	<4	4 (0.8)
Other forms of heart disease	<4	15 (7.5)	<4	4 (6.7)	<4	25 (5.1)
Cerebrovascular disease	<4	4 (2.0)	5 (3.5)	<4	<4	13 (2.6)
Diseases of veins and lymphatics	<4	0 (0.0)	<4	0 (0.0)	<4	<4
Pneumonia and pleurisy (n, %)	0 (0.0)	14 (7.0)	5 (3.5)	6 (10.0)	<4	27 (5.5)
Pneumonia and influenza	0 (0.0)	4 (2.0)	<4	0 (0.0)	0 (0.0)	6 (1.2)
COPD and allied conditions	0 (0.0)	0 (0.0)	<4	<4	0 (0.0)	4 (0.8)
Other diseases of respiratory system	0 (0.0)	13 (6.5)	5 (3.5)	6 (10.0)	<4	26 (5.3)
Gastrointestinal (n, %)	<4	5 (2.5)	0 (0.0)	<4	0 (0.0)	9 (1.8)
Liver (n, %)	<4	<4	<4	<4	<4	9 (1.8)
Renal (n, %)	<4	4 (2.0)	<4	5 (8.3)	0 (0.0)	13 (2.6)
Edema (n, %)	0 (0.0)	0 (0.0)	0 (0.0)	0 (0.0)	0 (0.0)	0 (0.0)
Metabolic alteration (n, %)	<4	16 (8.0)	14 (9.9)	<4	0 (0.0)	32 (6.5)
Years in treatment (mean, SD)	1.7 (1.7)	2.5 (1.6)	2.6 (1.6)	1.4 (1.2)	1.5 (1.2)	2.2 (1.6)

Supporting Table S4. The occurrence of comorbidities during treatment in patients in 3rd line stratified by TKI agents

	Imatinib	Dasatinib	Nilotinib	Bosutinib	Ponatinib	Overall
N. of patients	38	23	27	24	32	144
Hypertension (n, %)	<4	<4	<4	0 (0.0)	<4	9 (6.3)
Cardiovascular (n, %)	0 (0.0)	<4	<4	0 (0.0)	<4	5 (3.5)
Ischemic heart disease	0 (0.0)	<4	<4	0 (0.0)	<4	4 (2.8)
Diseases of pulmonary circulation	0 (0.0)	0 (0.0)	0 (0.0)	0 (0.0)	0 (0.0)	0 (0.0)
Other forms of heart disease	0 (0.0)	<4	0 (0.0)	<4	<4	<4
Cerebrovascular disease	0 (0.0)	<4	0 (0.0)	0 (0.0)	<4	<4
Diseases of veins and lymphatics	0 (0.0)	0 (0.0)	0 (0.0)	0 (0.0)	0 (0.0)	0 (0.0)
Pneumonia and pleurisy (n, %)	0 (0.0)	<4	0 (0.0)	<4	<4	5 (3.5)
Pneumonia and influenza	<4	0 (0.0)	0 (0.0)	<4	<4	<4
COPD and allied conditions	<4	0 (0.0)	0 (0.0)	0 (0.0)	<4	<4
Other diseases of respiratory system	0 (0.0)	<4	0 (0.0)	<4	0 (0.0)	<4
Gastrointestinal (n, %)	0 (0.0)	<4	0 (0.0)	<4	0 (0.0)	<4
Liver (n, %)	<4	0 (0.0)	0 (0.0)	0 (0.0)	<4	<4
Renal (n, %)	0 (0.0)	0 (0.0)	0 (0.0)	0 (0.0)	0 (0.0)	0 (0.0)
Edema (n, %)	0 (0.0)	0 (0.0)	0 (0.0)	0 (0.0)	0 (0.0)	0 (0.0)
Metabolic alteration (n, %)	0 (0.0)	<4	4 (14.8)	0 (0.0)	<4	6 (4.2)
Years in treatment (mean, SD)	1.9 (1.7)	2.4 (1.7)	2.5 (1.9)	1.1 (0.9)	1.5 (1.3)	1.9 (1.6)

Supporting Table S5. Mean daily dosages

	2nd line cohort Mean daily dosage (mg)	3rd line cohort Mean daily dosage (mg)	Label recommended, SMPC (mg/die)
Bosutinib	340.70	382.16	500*
Dasatinib	92.69	81.42	100
Imatinib	395.55	481.51	400
Nilotinib	609.19	656.44	800*
Ponatinib	31.81	37.88	45

* *recommended dosage starting form the second-line treatment*
